# Lifestyles and Academic Stress in University Students of Health Sciences: A Mixed-Methodology Study

**DOI:** 10.3390/healthcare12141384

**Published:** 2024-07-11

**Authors:** Yolanda E. Salazar-Granizo, Cesar Hueso-Montoro, Rafael A. Caparros-Gonzalez

**Affiliations:** 1 Doctorate Program in Clinical Medicine and Public Health, University of Granada, 18016 Granada, Spain; 2 Instituto de Investigación Biosanitaria (ibs-GRANADA), 18071 Granada, Spain; chueso@ujaen.es (C.H.-M.); rcg477@ugr.es (R.A.C.-G.); 3 School of Nursing, Faculty of Health Sciences, National University of Chimborazo, Riobamba 060101, Ecuador; 4 Faculty of Health Sciences, University of Jaén, 23071 Jaén, Spain; 5 Center for Mind, Brain and Behavior Research (CIMCYC), 18071 Granada, Spain; 6 Faculty of Health Sciences, University of Granada, 18016 Granada, Spain

**Keywords:** lifestyle, academic stress, online education, pandemic

## Abstract

The global health emergency generated by the COVID-19 pandemic (caused by the SARS-CoV-2 virus) led to the implementation of extraordinary measures such as confinement and isolation in many countries to mitigate the spread of the virus. (1) This study analyzes the lifestyles and academic and perceived stresses of university students of health sciences during the period of online learning due to the COVID-19 pandemic. The relationship between lifestyles and academic stress was examined. (2) A parallel mixed-method convergent study was conducted, with a correlational non-experimental design. Quantitative and qualitative data were collected and analyzed in parallel, with parametric and nonparametric testing for quantitative data and Miles and Huberman’s approach to qualitative analysis. The qualitative findings complemented the quantitative results. The number of students who participated in this study was 2734, from six programs in health, nursing, medicine, clinical laboratory, physiotherapy, dentistry, and clinical psychology at the University of Chimborazo, Ecuador. (3) Overall, the health science students had “Unhealthy or health-compromising lifestyles”, medical students being the ones who have healthier lifestyles. However, more than 80% experienced and perceived stress during the period of online learning and social isolation due to the pandemic, women being the ones who experienced it at a higher level. (4) The online learning modality during the COVID-19 pandemic modified lifestyles and generated stress in health science students, due to changes in daily routines, sedentary lifestyle, and stress, as a result of social isolation. Therefore, the students prefer face-to-face teaching, perceived as enabling more enriching interactions with their teachers and peers and the opportunity to develop essential practical skills in their health practice.

## 1. Introduction

The global spread of the coronavirus disease (COVID-19), first reported in December 2019, was declared a public health emergency of international concern in January 2020 by the World Health Organization (WHO) and subsequently categorized as a pandemic in March 2020 [1]. Traditional face-to-face teaching moved to online learning methods; the United Nations Educational, Scientific, and Cultural Organization, mentions that 1600 million students in the world adopted homeschooling during the pandemic [2].

The direct impact on physical and psychological health posed by the pandemic, in addition to the changes generated in social, cultural, economic, and educational activities, due to isolation, confinement, and social distancing measures represent a serious threat to people worldwide [3,4]. Yücel and Yücel [5] identified lifestyle modifications during the pandemic, such as changes in dietary habits, physical activity, and increased sedentary behavior due to the use of digital tools and social media [6]. The stressful events resulting from the COVID-19 pandemic generated uncertainty, fear, and anxiety in a large part of the population [7].

The COVID-19 pandemic changed the lifestyles and academic activities of students around the world, especially those in health disciplines [8]. With the aim of preventing the spread of the virus, governments implemented restrictions such as social distancing, home confinement, travel limitations, and the closure of sports and recreational facilities [9,10]. There was also a shift to remote learning modalities due to restrictions in access to universities, which had a considerable impact on education and community life and increased stress levels [11]. Undergraduate health science students faced numerous challenges during the COVID-19 pandemic that altered their lifestyles [12]. Academic and clinical demands affected their physical and mental well-being, increasing anxiety and stress due to their exposure to emotionally challenging situations and the need to adapt to new responsibilities [13].

Stress is defined as a state of physical and psychological arousal that arises when external demands exceed a person’s ability to cope, requiring adaptation or behavioral change [14]. As a biological regulatory mechanism, cortisol is released to adjust the magnitude of responses to stressors of various natures, whether physical or psychological [15]. High levels of stress are among the most significant toxic factors and are associated with the development of diseases [16]. An individual’s mental health can be adversely affected by stress if it persists for a prolonged period or reaches a certain level of intensity [17]. University students experienced anxiety and depression, as well as heightened academic stress, during the pandemic, potentially causing adverse effects on mental health in this population [18].

The transition to the online learning modality, motivated by safety reasons [19], led to the suspension of training activities in healthcare units and exposure to risks of infection during clinical practices [20], among other significant changes. These changes had an impact on both the lifestyles and education of health students [21], influencing their academic performance [22] and even the continuity of academic training [23]. In response to this situation, a self-directed/self-regulated learning approach [24] and the implementation of new teaching pedagogies [25] were required.

The rigorous study schedules resulting from curricular adjustments generated an increased workload, exerting additional pressure on students. The transition to home-based education complicated the separation of academic and personal life and limited social interaction in group projects or extracurricular activities, potentially causing anxiety, stress, and feelings of loneliness [26]. Stringent evaluations and the pressure to obtain high grades constituted academic stressors for students in health-related programs [27]. Moreover, concerns and uncertainty about not achieving professional competencies due to the interruption of clinical practices, graduation, and career projects contributed to higher levels of stress and anxiety in the students’ adaptation process [26,28].

Studies have reported changes in lifestyle and learning modalities among university students caused by the pandemic. However, it is important to relate these changes to the generation of academic and perceived stress, considering their impact on six health programs with different graduation profiles and competencies to be developed in their academic training–significant aspects for student well-being and educational success. Additionally, it should be considered that the pandemic may trigger emotional distress and social disability, even after the disease has been eradicated [29].

The present study aimed to analyze the lifestyles, academic stress, and perceived stress of university health science students in the context of the online learning modality during the COVID-19 pandemic, as well as the relationship between lifestyles and academic stress. The hypotheses were as follows: (1) The online learning modality, a consequence of the COVID-19 pandemic, modified lifestyles and generated academic and perceived stress in health science students. (2) Changes in lifestyle are related to the generation of academic and perceived stress. The research questions were as follows: Which dimensions of lifestyle were modified in health science students during the pandemic? Which dimensions generated academic stress in health science students during the pandemic? Are lifestyle changes related to academic stress?

## 2. Materials and Methods

### 2.1. Study Design and Procedures

A correlational study was conducted with a convergent parallel mixed approach. The results of the qualitative analysis complemented those of the quantitative approach. 

Information about the study, including the project details, research objective, informed consent process, and anonymization of student data to protect privacy and confidentiality, was provided in the participant information document sent via email prior to questionnaire administration. Furthermore, synchronous activities (video conferences) were conducted with the authors’ participation, during which they shared project information and encouraged students from each degree program to participate.

### 2.2. Study Population

All students of the nursing, medicine, clinical laboratory, physiotherapy, dentistry, and clinical psychology programs at the Faculty of Health Sciences of the National University of Chimborazo (UNACH-Ecuador) (N = 2880) who were enrolled in the last period of online learning (18 April 2022, to 4 August 2022) necessitated by the COVID-19 pandemic were invited to participate; the invitations were sent via email and the university’s Academic Platform (SICOA).

All students were invited to participate, obtaining responses from a large number of individuals, so convenience sampling was used; data were requested from the complete set of students during the period of interest, which ensured the inclusion of almost all relevant individuals within the target population, providing a comprehensive understanding of the phenomena under investigation. After informative meetings and informed consent was obtained, data were collected online, and responses were finally obtained from n = 2734 students. 

### 2.3. Instruments

To assess lifestyle, the Lifestyle Profile questionnaire (PEPS-I) by Nola Pender was used, which quantitatively measures the level of lifestyle using 48 items in 6 dimensions: nutrition, exercise, health responsibility, stress management, interpersonal support, and self-actualization [30]. The items are rated using a four-point Likert scale (never = 1, sometimes = 2, frequently = 3, routinely = 4). The evaluations range from 48 to 192, with higher evaluations indicating a healthier lifestyle [31]. The instrument was validated by León-Reyna et al. [32] for the Peruvian population, with a Cronbach’s alpha of 0.96.

The sources of stress, physical, psychological, and behavioral reactions, as well as coping strategies, were evaluated using the Systemic Cognitivist Inventory for the Study of Academic Stress, with 21 items rated on a five-point Likert scale (except for the dichotomous filter item) (never = 1, rarely = 2, sometimes = 3, almost always = 4, always = 5) and a score between 21 and 105. Higher scores indicate higher academic stress. The instrument was validated by Ruiz-Camacho and Barraza-Macias [33] for the Spanish population, with a Cronbach’s alpha of 0.85.

Perceived stress was measured using the Perceived Stress Scale, which includes 14 responses that explore the feelings and thoughts experienced over the past month. Items 1, 2, 3, 8, 11, 12, and 14 assess perceived stress, measured through a five-point scale with 0 = never, 1 = almost never, 2 = occasionally, 3 = often, 4 = very often; items 4, 5, 6, 7, 9, 10, and 13 measure coping with perceived stress measured through a five-point scale with 4 = never, 3 = almost never, 2 = occasionally, 1 = often, 0 = very often; scores are for each item [34]. Higher scores indicate a greater perception of stress, and scores above 15 are indicative of the presence of high levels of stress [35]. The instrument was validated by Larzabal-Fernandez and Ramos-Noboa [36] for the Ecuadorian population, establishing a reliability expressed as Cronbach’s alpha internal consistency (α) between 0.805 and 0.811. 

In addition, four open-ended questions were added to learn about the students’ lifestyles and their relationship with stress, lifestyle changes during the COVID-19 pandemic, academic stress, and their learning modality preference, which allowed for deepening the understanding of the experiences and perspectives of the participants in the specific context of their training, from a qualitative perspective. 

Demographic questions collected information on the participants’ age, sex, program, semester, academic average, internet access, technological equipment (computer, smart phone, laptop, tablet), and technological equipment conditions.

### 2.4. Data Analysis 

Quantitative data processing was performed using the SPSS Statistical Package for Social Sciences, version 24.0; (IBM Corporation: Armonk, NY, USA). The descriptive data are presented as the frequency and measures of central tendency. Analyses of the variables are shown using, as appropriate, chi-square relationships (χ^2^), independent t-Student comparisons, and one-way ANOVA, followed by multiple Games–Howell and Tukey comparisons. In addition, covariates that may influence the results (ANCOVA) were added. Regression models were used to calculate significant predictors of healthy lifestyles and those that increased academic stress. Demographic variables that were significantly related to lifestyles and academic and perceived stresses were introduced into the regression models. The combined use of parametric and non-parametric approaches enabled a more comprehensive analysis of the data, leveraging the strengths of each type of test and adequately addressing the different nature of the variables involved to achieve a more consistent and enriched interpretation of the results, maximizing the explanatory potential in the research.

Qualitative inputs were obtained voluntarily among the participants and subjected to analysis based on the scheme of Miles and Huberman [37]. The qualitative data were analyzed in three simultaneous streams—data condensation, visualization, and extraction and verification of conclusions—which allowed for maintaining a continuous approach between the ideas and experiences of the participants. We proceeded with the condensation of the contributions into thematic content units, identifying, simplifying, and classifying the elements to establish categorization and coding. Variables, patterns, and themes with similar contents were identified and grouped with the use of matrices by career, semester, and sex. The grouping of similar ideas was carried out using the software MAXQDA, version 2020 (VERBI Software GmbH: Berlin, Germany). Results were obtained, and the reached conclusions were verified, checking their representativeness, analyzing the effects of the researcher, triangulating them with multiple data sources, and finally considering the quality and robustness of each piece of evidence that supports a conclusion. In addition, study participants commented on the findings to ensure the researchers accurately captured their perspectives and experiences.

### 2.5. Ethical Considerations

The research from which this article was derived was analyzed and approved by the Committee on Ethics in Research on Human Beings [Resolution No. CEISH UCACUE-052], a committee recognized by the Ministry of Public Health of Ecuador. 

To protect the confidentiality of the data provided in the research, an anonymization process was used to minimize the identification of students.

## 3. Results

### 3.1. Descriptive Findings

The population was N = 2880 university students of Health Sciences at the University of Chimborazo, Ecuador. The response rate was 94.9% (n = 2734) enrolled students, of whom 70.9% (n = 1938) were female. A total of 99.4% were of Ecuadorian origin (n = 2717), and 90.9% self-identified as mestizos (n = 2485), with the highest percentage corresponding to a career in medicine (24.8%; n = 678). A total of 78.9% students reported having good internet access (n = 2156), and 44.6% (n = 1218) had more than one technological device for academic activities during the period of online learning. The mean age of the students was M = 21.6 years (SD = 2.52), and the grade point academic average was M = 8.24 (SD = 0.73) on a scale of zero to ten (Table 1).

### 3.2. Quantitative Results

The analysis of the lifestyles, academic stress, and perceived stress of health students identified:

Lifestyles: The students present an overall mean lifestyle of M = 113.62 (SD = 23.24), indicating Unhealthy or health-compromising lifestyles. The self-actualization dimension, which analyzes actions aimed at promoting personal development and satisfaction, presented the highest average score, M = 38.36 (SD = 8.42); the dimensions exercise, stress management, and nutrition had a lower mean score M = 10.37 (SD = 3.02); M = 12.97 (SD = 3.28); M = 14.54 (SD = 3.46), respectively. The results of Student’s *t*-test, with a significance level set at *p* = 0.05, showed that men had a significantly higher mean lifestyle score of M = 117.51 (SD = 24.50) compared to that of women, M = 112.02 (SD = 22.52).

The relationship between lifestyles and perceived stress based on the chi-square test was significant at *p* = 0.05. This shows that students who perceived that they never need to face stressful situations had a lower percentage of healthy lifestyles (0.2%) compared to those who perceived that they must face them from time to time (3.4%). 

A significant difference (ANOVA) was found in the students’ lifestyles based on their degree (F (5; 2728) = 3.20, *p* = 0.007). The post hoc study showed that the medical students had significantly healthier lifestyles than the dental students (mean difference = −4.70, 95% CI = [−8.59, −0.82], *p* = 0.006). Additionally, the corrected model (ANCOVA) (F = 3.25, *p* = 0.06) indicates that there were significant differences among the students’ lifestyles in the variables of career path, semester, and their interaction; these variables met the assumptions of independence, normality, homoscedasticity, linearity, and homogeneity. The post hoc analysis indicates that, compared to the nursing program, students of the medicine (M = 116.332) and dentistry (M = 111.388) programs had significantly different lifestyles. Medical students had healthier lifestyles by 4.94 points on average (95% CI (1.01 to 8.86)) compared to dentistry students, who had less healthy lifestyles, with −4.94 points (95% CI (−8.86 to −1.01)) on average (Table 2).

Academic Stress: This is the pressure and strain experienced by students due to their academic commitments. The mean academic stress was M = 59.03 (SD = 25.85), indicating that students have a moderate level of stress. The results of each of the systemic processual components of stress and the general stress level are interpreted using three cut-off points at the 33rd (mild level of stress), 66th (moderate level of stress), and 100th (severe level of stress) percentiles. Additionally, 88.40% of the students (n = 2416) indicated that they had been worried or nervous and had devised coping measures, suggesting that they had some degree of academic stress. It was verified that 63.7% (n = 1740) were exposed to stressors; 63.5% (n = 1735) reported having “sometimes” or “almost always” experienced symptoms of academic stress. Female students (M = 60.74; SD = 25.17) experienced higher levels of academic stress compared to males (M = 54.87; SD = 27.01; *p* = 0.05) and higher perceived stress (male: M = 14.08 vs. female: M = 15.11, *p* < 0.01). 

In the present analysis, significant differences (ANOVA) were found between academic stress and career path (F = 7.54; *p* = 0.001). Dentistry students had higher levels of academic stress. The corrected ANCOVA model examined the relationship between academic stress and career/semester (F = 7.44; *p* = 0.001). The post hoc model shows that the medical and dentistry students had significantly (*p* = 0.001) higher levels of academic stress than the physiotherapy students. In addition, the dentistry students had significantly higher levels (*p* = 0.024) of academic stress than the clinical psychology students. Internet access (F = 11.10; *p* = 0.001) and the state of the students’ technological equipment (F = 7.677; *p* = 0.001) were also significantly related to academic stress, with students with “poor” access having higher levels of academic stress.

With the application of the logistic regression model, it was determined that the students’ ages and their perceived coping were significantly related to their academic stress. For each unit increase in student age, the odds ratio (OR) of academic stress increased by 1.2 times (95% CI: 1.08 to 1.17); their perceived coping was significantly related to their academic stress, with an OR of 1.83 (95% CI: 1.44 to 2.33; Table 3). 

Perceived Stress: This is individuals’ predisposition to feel stressed. According to the test results, there was a significant difference (*p* < 0.001) between the students’ perceived stress and their sex (Student’s *t*-test). Female students (M = 15.54; SD = 5.34) reported higher levels of stress perception than male students (M = 14.08; SD = 5.15). Single women and men (48.2% and 48.6%) reported similar levels of stress, while married and common-law marriage students reported lower perceived stress (*p* = 0.005).

The ANOVA results indicate that there were significant differences in the levels of perceived stress among the different programs (F (5; 2728) = 3.20, *p* = 0.001). The multiple comparisons show that the nursing students had a lower level of perceived stress than the dentistry students (*p* = 0.006). The corrected ANCOVA model showed significant differences among the variables career path and career level and their interaction (F = 9.610; *p* = 0.001). In the post hoc analysis, it was found that the physiotherapy students had a significantly lower score for perceived stress compared to their peers in the career pathways of nursing, medicine, clinical laboratory, and clinical psychology (*p* < 0.001). In addition, students in the dentistry program had a significantly higher score for perceived stress compared to students in the physiotherapy program (*p* < 0.001). 

Regarding perceived coping, most students (67.5%; n = 1846) reported using coping strategies “sometimes” or “almost always”, the medical students had the highest percentage of perceived coping “from time to time” (12.3%). Regarding the academic average variable, it was observed that perceived coping was related to an excellent (9–10), very good (8–8.9), and good (7–7.9) academic average, but not to failure (<7).

### 3.3. Qualitative Results

**Figure 1:**
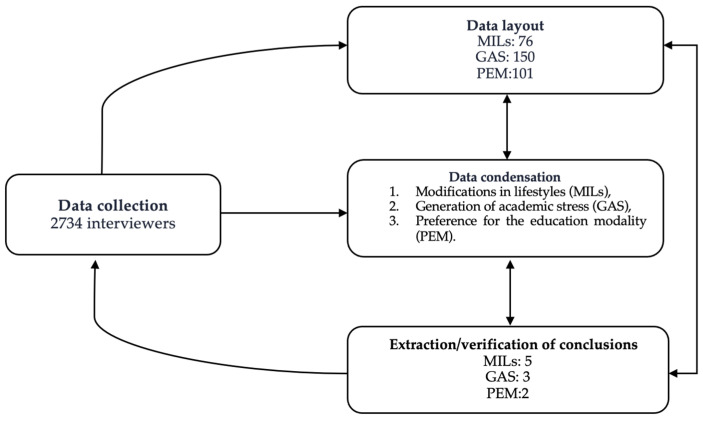
Qualitative analysis methodology diagram.

The findings reveal the opinions of the students from the six career paths at the Faculty of Health by semester and sex. Through their narratives, they revealed their concerns about the changes caused in their personal and academic lives during the period of online learning due to the COVID-19 pandemic. Of the contributions made, three themes were defined and codified: (1) modifications in lifestyles (MILs), (2) the generation of academic stress (GAS), and (3) preference for the education modality (PEM). Answers that describe the three identified topics were chosen, and conclusions were generated and verified, representing the feelings of most of the students (Figure 1).

MILs: Students mentioned that the virtual modality made it difficult to maintain a daily academic routine, pay attention to classes, and develop practical skills. The lack of physical activity and a sedentary lifestyle led to weight gain and mental and emotional health problems, such as stress, depression, and anxiety. Social isolation had an impact on interaction skills with others. Economic problems, job losses, and decreased income affected the financial stability of the students and their families. Some students found the virtual modality to be useful because they were able to develop new hobbies or interests and share them with their families.

*“I feel that the social aspect was in decline because it was very difficult to socialize with people, and education changed completely.”*

(Male nursing student)

*“Personally, the isolation affected my psychological side as it took me a while to get used to that way of life.”*

(Female medical student)

GAS: The education modality generated academic stress, especially due to the lack of face-to-face practice, the overload of tasks, difficulties connecting to the internet, and the lack of time for evaluations.

The presence of physical symptoms, such as low back pain and visual problems, related to the prolonged use of electronic devices, influenced the generation of stress. Students experienced anxiety, depression, panic attacks, low self-esteem, and lower academic performance, and a decreased ability to concentrate, social isolation, difficulty studying, and mood swings were generators of stress.

*“It mainly influenced the concern about not having a stable internet network, which resulted in sometimes not being able to attend classes or not being able to fully understand a class due to the failure in connectivity.”*

(Male physiotherapy student)

*“I have a lot of social anxiety, embarrassment and too many nerves to be able to express myself freely and my academic performance is what depresses me the most every day.”*

(Female clinical laboratory student)

PEM: Students reported a preference for the face-to-face study modality, mainly attributing their choice to a perception of higher-quality teaching with direct interactions with their teachers and classmates, the absence of distractions, and the opportunity to practice and develop essential skills and abilities in their respective areas and make relevant observations. On the other hand, some students expressed a preference for the mixed or virtual modality, although to a lesser extent, mainly due to family and economic situations.

*“Face-to-face because it forces me to stay busier away from situations and put aside other thoughts, concentrating on my studies.”*

(Male dentistry student)

*“Face-to-face because I feel that everything, they give me can be done in a certain practical way and there won’t be as many complications as there were virtually, this thing that the internet went out or you couldn’t clearly hear what they were teaching us.”*

(Female clinical psychology student)

## 4. Discussion

The online learning modality and the period of mandatory social isolation due to the COVID-19 pandemic modified the lifestyle of and generated academic and perceived stress among health science students at the National University of Chimborazo. The analyses focused on the modifications in lifestyle and the generation of academic and perceived stress. Previous research, such as studies by Yücel and Yücel [5], Souza et al. [38], and Suliman et al. [19], has mentioned that increased time spent using technological equipment, sleep disturbances, and a sedentary lifestyle caused an increase in and perception of weight gain, as well as anxiety, stress, and frustration during the transition to remote learning [39]. These results are similar to those found in the current research, which reported unhealthy or health-compromising lifestyles in the dimensions of exercise, stress management, and nutrition. This evidence suggests that changes in lifestyles may be responses to the aggravating factor of the COVID-19 pandemic, a crisis that resulted in significant psychological burdens [40].

In university health students, the dimensions of nutrition and exercise were altered by changes in their lifestyle due to mandatory social isolation and online education, in-creasing their body weight and generating mental and emotional health problems, such as stress, depression, and anxiety. Chick et al. [41] discussed how health behaviors, including inadequate eating habits, less physical activity, and sedentary behavior, caused symptoms of insomnia, depressive symptoms, and anxiety. The exercise dimension exhibited the lowest mean value among all dimensions, indicating that it was the most significantly impacted.

Considering the sex of the participants, the male students presented healthier life-styles compared to the women during the mandatory social confinement. The male students were more active and presented better eating habits than their female peers; however, Sultana et al. [42] concluded that the male participants had unhealthy lifestyles, their physical activity was significantly reduced, and they were highly prone to tobacco and drug use during the COVID-19 pandemic. 

Students in health sciences experienced academic stress related to learning processes and the presence of stressors, such as a high level of demand from their teachers, overload of work, difficulties connecting to the internet, and the lack of time for evaluations. This generated a reluctance to perform tasks and concerns about the scope and achievement of practical learning results due to not being able to attend hospitals and laboratories, and online education did not provide opportunities to practice. These results are consistent with those found by Lovrić et al. [43], and the lack of hands-on training opportunities affected students’ motivation, concentration, and general well-being; the online learning modality was not engaging or enjoyable, and opportunities to ask questions were limited [44]. Additionally, Bdair [45] discussed how the drastic shift to online learning was a stressful experience for students, with increased or more difficult tasks. Masha’al et al. [46] referenced students’ fears that online teaching may compromise their clinical competence and confidence. 

The students of health sciences who participated in this study were susceptible to changes in the learning process. Their training depends largely on interpersonal interaction and contact with people, skills that are developed in training and pre-professional practice, in addition to the strengthening of clinical skills. Saddik et al. [47] mentioned that medical students experienced lower levels of anxiety during the pandemic than dental students, and women reported higher levels of academic stress [48]. These findings are consistent with the results obtained from the National University of Chimborazo health students, where dentistry students and female students experienced higher levels of aca-demic stress compared to their counterparts. 

Changes in the learning context (online modality) are related to the generation of stress, such that students prefer face-to-face education because of the advantages for their training, mainly attributing their choice to the perception of higher-quality teaching being associated with direct interactions with their teachers and classmates, the absence of distractions, the opportunity to practice and develop essential skills and abilities in their respective areas and make relevant observations. As mentioned by Bdair [45], conventional, face-to-face learning is still the preferred modality [18,49]. Online learning affected students’ perception of readiness for practice. However, some cited advantages of hybrid education due to their family and economic situations, as reported by Suliman et al. [19]. Some students also reported feeling more independent and self-directed in their learning using the online education modality. 

Lifestyle modifications are not directly related to the generation of academic stress; however, they have been altered due to changes in the global context caused by the COVID-19 pandemic. This situation has revealed weaknesses, limitations, and opportunities for improvement in health education. The online learning modality likely restricted the development of practical competencies and skills, as well as demonstrated difficulties in adaptation and time management when working from home. Nevertheless, the rapid transition to remote learning testifies that it is possible to seek innovative mechanisms that support the health education process in crisis situations, which will ensure comprehensive training to safely address patient needs in diverse contexts.

### Limitations and Suggestions for Future Studies

The findings underscore the necessity for future multi-institutional research to explore the long-term effects of the pandemic on student well-being and academic outcomes. By broadening the scope of the study to encompass diverse disciplines and considering novel variables, future research may provide a more comprehensive understanding of the pandemic’s impact on university students. Furthermore, the utilization of longitudinal designs can aid in establishing causal relationships and assessing the enduring impact of the pandemic on students’ lives. 

The insights gleaned from this study and future research can inform the development of targeted interventions and support strategies to promote the well-being and academic success of university students during challenging times.

The findings of this study will inform the development of strategies for effective interventions that enhance academic adaptability and contingency plans that promote students’ psychosocial well-being. These strategies should incorporate technical support and appropriate training for personnel in higher education institutions. Curricula should be flexible and ensure that students acquire essential technological competencies and include methodologies that facilitate the autonomous consolidation of practical learning outcomes and synchronous peer-to-peer collaborative learning. Comprehensive wellness programs within institutions should provide holistic support addressing physical, emotional, and mental health needs through ongoing accompaniment services. These services should prioritize cases requiring urgent attention for both students and faculty.

## 5. Conclusions

Health science students experienced changes in their lifestyles, especially in exercise, stress management, and nutrition, related to the mandatory social confinement due to the COVID-19 pandemic. The change in the learning modality forced them to spend long hours using technological equipment, which influenced the changes recorded.

The students expressed concern and experienced academic stress due to the changes in the modality of studies. These changes involved technical problems due to the quality of the technological equipment, long hours of online work, and a perception of a decrease in the quality of practical training and the interaction with teachers, mainly due to the lack of relationship with patients, a fundamental axis in health training.

Most of the students preferred the face-to-face modality for their training, as they perceived it to be of higher quality, mainly because of the opportunity to carry out internships and develop essential skills in their respective areas. However, some students preferred the blended or virtual modality due to economic or personal factors.

The COVID-19 pandemic, an emerging situation with a global impact, has caused changes in academic, social, economic, and health contexts, which has led to changes in lifestyles and has generated academic stress in university students of health sciences.

## Figures and Tables

**Table 1:** Demographic data (n = 2734).

Variable	Frequency	%	M (SD)
Age *			
18–25	2449	89.5	21.6 (2.52)
26–32	277	10.1	
33–40	6	0.2	
41+	2	0.1	
Sex			
Women	1938	70.9	
Man	796	29.1	
Programs			
Nursing	393	14.37	
Medicine	678	24.80	
Physiotherapy	410	15.00	
Clinical Laboratory	315	11.52	
Dentistry	562	20.56	
Clinical psychology	376	13.75	
Semester			
First	342	12.5	
Second	233	8.5	
Third	256	9.4	
Fourth	301	11.0	
Fifth	365	13.4	
Sixth	281	10.3	
Seventh	237	8.7	
Eighth	305	11.2	
Nineth	143	5.2	
Tenth	165	6.0	
Rotating Internship	106	3.9	
Academic average *			
Excellent (9–10)	399	14.6	8.24 (0.73)
Very Good (8–8.9)	1583	57.9	
Good (7–7.9)	553	20.2	
Failed (<7)	199	7.3	
Internet access			
Excellent	264	9.7	
Well	2156	78.9	
Bad	314	11.5	
Technological equipment			
Mobile phone	185	6.8	
Laptop	622	22.8	
Desktop computer	703	25.7	
Tablets	6	0.2	
More than one	1218	44.6	
Technological equipment conditions			
Excellent	352	12.9	
Good	2194	80.2	
Bad	188	6.9	

* The variables are presented in the table in classes with intervals to facilitate visualization. However, the statistical analyses were performed using the original data collected as a discrete quantitative variable (age) and continuous quantitative variable (grade point academic average). Note: % = percentage; M = mean; SD = standard deviation.

**Table 2:** Lifestyle association ANOVA/interaction ANCOVA (n = 2734).

Variable Lifestyle	Association ANOVA			Intersection PROGRAMS + SEMESTER * ANCOVA		
	M (SD)	F (*df*)	Post Hoc CI 95%	M	F	Post Hoc CI 95%
Programs						
Nursing	114.76 (23.21)	3.20 (5); *p* = 0.007 **		114.241 ^a^	3.25; *p* = 0.006 **	
Medicine	116.13 (23.72)		4.70 (0.82 to 8.59) *p* = 0.006 **	116.332 ^a^		4.94 (1.01 to 8.86) *p* = 0.003 **
Physiotherapy	112.06 (23.62)			112.110 ^a^		
Clinical Laboratory	113.22 (21.87)			113.488 ^a^		
Dentistry	111.42 (23.23)		(−4.70) (−8.59 to 0.82) *p* = 0.006 **	111.388 ^a^		(−4.94) (−8.86 to −1.01) *p* = 0.003 **
Clinical Psychology	113.21(22.78)			113.025 ^a^		

** The difference in means is significant at the 0.02 level; * Corrected model; ^a^: Adjusted mean.

**Table 3:** Academic stress association ANOVA/interaction ANCOVA (n = 2734).

Variable Academic Stress	Association ANOVA			Intersection PROGRAMS + SEMESTER * ANCOVA		
	M (SD)	F (*df*)	Post Hoc CI 95%	M	F	Post Hoc CI 95%
Programs						
Nursing	59.75 (24.92)	7.54 (5; 2728) *p* = 0.001 **	6.70 (1.47 to 11.94) *p* = 0.004 **	59.045 ^a^	7.44 *p* = 0.001 **	
Medicine	60.38 (25.09)		7.33 (2.63 to 12.0) *p* = 0.001 **	60.965 ^a^		7.62 (2.90 to 12.35) *p* = 0.001 **
Physiotherapy	53.04 (27.02)		(−9.51) (−14.4 to −4.59) *p* = 0.001 **	53.337 ^a^		(−9.33) (4.45 to −4.45) *p* = 0.001 **
Clinical Laboratory	59.30 (24.62)			58.896 ^a^		
odontology	62.56 (25.81)		9.51 (4.59 to 14.43) *p* = 0.001 **	62.672 ^a^		9.33 (1.01 to 14.21) *p* = 0.001 **
Clinical Psychology	56.87 (26.81)		(−5.68) (−10.71 to −0.66) *p* = 0.016 **	57.286 ^a^		(−5.38) (−10.39 to −0.37) *p* = 0.024

** The difference in means is significant at the 0.02 level; * Corrected model; ^a^: Adjusted mean.

## Data Availability

The data used during this study are available from the corresponding author, upon request by email.
